# Healthy lifestyle mediates the association between health locus of control and life satisfaction among college students in Hubei, China: during the normalization stage of COVID-19 epidemic prevention and control

**DOI:** 10.1186/s13690-023-01145-9

**Published:** 2023-07-24

**Authors:** Ying Mei, Yuzhou Zhang, Jincong Yu, Xuemei Tang, Wenzhen Li

**Affiliations:** 1 School of Physical Education, Jianghan University, 8 Sanjiaohu Road, Wuhan, 430056 China; 2 School of Marxism, Central China Normal University, 152 Luoyu Road, Wuhan, 430079 China; 3 Department of Student Affairs, Zhongnan University of Economics and Law, 182 Nanhu Avenue, Wuhan, 430073 China; 4 Education and Counseling Center for Psychological Health, Zhongnan University of Economics and Law, 182 Nanhu Avenue, Wuhan, 430073 China; 5 Adolescent Psychology Development Institute, Zhongnan University of Economics and Law, 182 Nanhu Avenue, Wuhan, 430073 China; 6 School of Marxism, Wuhan Railway Vocational College of Technology, 1 Canglong Avenue, Wuhan, 430072 People’s Republic of China; 7 JC School of Public Health and Primary Care, The Chinese University of Hong Kong, Hong Kong, China

**Keywords:** College students, Health locus of control, Healthy lifestyle, Life satisfaction, Mediation

## Abstract

**Background:**

Previous studies have primarily focused on the relationships among the health locus of control (HLC), healthy lifestyle and life satisfaction of college students. However, little is known about the mediating mechanism of healthy lifestyle on the other two aspects. This study aims to address this issue.

**Methods:**

A total of 2394 students from six colleges in Hubei Province validly completed self-report questionnaires, including the Satisfaction with Life Scale (SWLS), Healthy Lifestyle Questionnaire for college students and Multidimensional Health Locus of Control Scale (MHLC), which covered three dimensions: internal HLC, powerful others HLC and chance HLC. Partial least squares structural equation modelling (PLS-SEM) was employed to analyses the hypothesized relationships in the path model, and a mediation analysis was used to verify the indirect relationships.

**Results:**

Healthy lifestyle and life satisfaction showed positive relations with both internal HLC and powerful others HLC, but a significant negative association with chance HLC. In addition, healthy lifestyle mediated the relationships of internal HLC, powerful others HLC and chance HLC with life satisfaction.

**Conclusions:**

Healthy lifestyle mediates the impact of HLC on life satisfaction. Students with high IHLC tend to develop a healthier lifestyle and have higher LS. Powerful others also exert positive effects in collectivist cultural backgrounds. Thus, colleges should give full play to the positive role of psychological health and physical education courses in improving students’ IHLC. Meanwhile, the positive guiding effect of powerful others should be stressed. Further, particular emphasis should also be placed on the peer influence, new media publicity functions, community intervention, and college systematic appraisal, especially during and after public health emergencies.

**Supplementary Information:**

The online version contains supplementary material available at 10.1186/s13690-023-01145-9.

**Table:** 

Text box 1. Contributions to the literature
• Healthy lifestyle mediates the relationship between health locus of control and life satisfaction.
• In collectivist cultural backgrounds, powerful others health locus of control would positively predict healthy lifestyle and life satisfaction.
• For college students, to reduce the negative impact of public health emergencies on life satisfaction, particular emphasis should be placed on the college psychological health and physical education courses, authorities and peer influence, new media publicity functions, community intervention, and college systematic appraisal functions.

## Introduction

Numerous studies have demonstrated that under the influence of the COVID-19 pandemic, various social groups have shown significant increases in mental distress, which has negatively affected their well-being and life satisfaction (LS) [1–4]. Compared with other social groups, when faced with the COVID-19 pandemic, college students may have lower mortality and less physical vulnerability [5]. However, due to their psychological vulnerability, they may be challenged by psychological issues, such as a sense of social isolation, fear, stress and mental health disorders [6]. These issues mainly caused by epidemic prevention measures such as periodic lockdowns, remote online learning [7] and have led to dramatic decreases in their LS levels [8]. Since the health and well-being of individuals during the COVID-19 pandemic are mostly determined by their own behaviours and beliefs [7], it is necessary to conduct relevant research from behaviour and cognitive perspectives.

LS is a cognitive dimension of subjective well-being that can be defined as individuals’ level of fulfillment in their current life and personal desire [9, 10]. For college students, LS has been identified as a predictor and indicator of functioning, comorbidities and clinical symptoms [11]. Individuals with higher LS are more likely to have social support and self-efficacy, and are more likely to continuously plan and consider future consequences [12, 13]. However, low LS may lead to psychological distress, depression, and anxiety [14, 15]. In addition, due to the large number of college students in Hubei Province, it is imperative to explore the factors affecting their LS during the COVID-19 pandemic.

### Theoretical background and hypothesis development

Health locus of control (HLC) is a derivation of locus of control, and has been developed into the Multidimensional Health Locus of Control (MHLC) scales by Wallston et al. [16]; the scales comprise one internal dimension (internal HLC, IHLC) and two external dimensions, namely, chance HLC (CHLC) and powerful others HLC (PHLC) [16]. IHLC measures the extent to which “individuals believe health outcomes are dependent on their own effort and ability”; CHLC measures the belief that one’s health status is closely related to chance, fate and luck; and PHLC represents a belief that one’s health status is largely determined by other powerful individuals such as physicians. HLC possesses cognitive-attribution characteristics and has been investigated as an important dynamic mechanism and a driver of LS development [17–20]. IHLC is positively associated with LS, and individuals with greater IHLC values are more likely to regard life changes as a challenge rather than a threat and are less likely to have health-related fears when confronted with sudden emergencies (e.g., COVID-19), which may enhance their level of psychological well-being [21–23]. CHLC is negatively related to LS, since individuals with higher CHLC values are more likely to experience higher levels of depression and lower psychological well-being [21]. Since they believe they cannot control their life; thus, negative emotion may occupy a dominant position [24]. However, according to different cultural backgrounds, both positive and negative correlations between PHLC and LS have been identified. In collectivist cultures, PHLC may be positively related to LS; thus, their relationship remains disputable [3, 25].

A healthy lifestyle is a collection of health-supporting behaviours and can be defined as “a living pattern that reduces the risk of serious illness or premature death” [26]. It can be specifically manifested by healthy dieting, physical activity, health responsibility, stress management and interpersonal relationships [27]. Previous studies have demonstrated that HLC is an antecedent influencing factor of healthy lifestyle development [28–30]. IHLC is generally associated with a healthy lifestyle, such as regular exercising and a healthy diet [28]. Individuals with higher IHLC values tend to have higher persistence in waiting for the materialization of their health investments [29]. External HLC, particularly CHLC, is directly related to unhealthy lifestyles such as smoking, alcohol consumption and unhealthy dieting [28, 30]. In addition, PHLC is negatively associated with healthy lifestyle in individualistic cultural backgrounds [29]. However, this relationship might be the opposite in collectivist cultures and remains to be further investigated and determined.

Previous research has also demonstrated that healthy lifestyle behaviours are important antecedent factors influencing LS [31, 32]. Accordingly, a healthy lifestyle is closely associated with happiness and well-being [33], and people can maintain their LS by cultivating a healthier lifestyle [34]. Since different groups evaluate their life quality with unique sets of criteria, the evaluation results of LS may show significant variation [35]. For college students, LS is one of the most important indicators of successful life adaptation and psychological health [36, 37] and can be evaluated from the perspective of healthy lifestyle [36]. Some studies have demonstrated this viewpoint indirectly, and concluded that college students with less study burden, good eating habits, regular lifestyles, regular physical activities, and good interpersonal relationships have higher LS level [38, 39]. Therefore, a healthy lifestyle is a vital factor related to LS.

In summary, the above studies have explored whether a healthy lifestyle is not only influenced by HLC but also has a vital effect on LS. In addition, Weiss and Larsen [40] showed that individuals with high IHLC appear more likely to engage in specific healthy behaviour, which may have a modifying effect on their life satisfaction. Therefore, based on this literature and the above hypotheses, it is obvious that HLC, healthy lifestyle, and LS are closely related, and healthy lifestyle might mediate the relationship between HLC and LS. However, within the scope of our cognition, there is no research to verify this mediating effect. Hence, this study aims to determine how healthy lifestyle mediates HLC and LS, and verify the direct relationships among the HLC, healthy lifestyle and LS of college students in collectivist culture. Based on the above literature, the following hypotheses are proposed:

- H1: IHLC is positively related to LS
- H2: CHLC is negatively related to LS
- H3: PHLC is positively related to LS
- H4: IHLC is positively related to Healthy lifestyle
- H5: CHLC is negatively related to Healthy lifestyle
- H6: PHLC is positively related to Healthy lifestyle
- H7: Healthy lifestyle is positively related to LS

- H8: Healthy lifestyle mediates the relationship between HLC and LS

## Methods

### Study design and participants

In this study, a survey was conducted with the purposive sampling method in four undergraduate colleges and two higher vocational colleges in Hubei Province from October to December, 2020. The participants were college freshman to senior college students who selected the Mental Health of College Students class. Participants were randomly assigned to form classes according to student ID by office of academic affairs, each class contains students from two or more majors. Overall, all participants came from different majors and different regions of China. In the process of issuing questionnaires, we mainly employed the well-trained psychology course teachers and college counsellors to provide a detailed introduction of the questionnaire contents. The questionnaires were distributed through SurveyStar, a Chinese online questionnaire platform. The invited students completed the questionnaires by clicking the exclusive link or scanning a specific QR code, and each mobile phone or computer could be used only once. In total, the questionnaires were distributed to 2933 college students. At the end of the survey, 2715 questionnaires were retrieved, yielding a response rate of 92.57%. Considering the validity of the data, only the questionnaires with an answering length of more than 260 s were retained. Finally, 2394 valid questionnaires were used for further study, for an effective rate of 88.18%.

## Measures

### Life satisfaction

LS was measured with the Satisfaction with Life Scale (SWLS) developed by Pavot et al. [41]; it is one of the most commonly used cognitive judgement methods to measure satisfaction with one’s life. To date, it has been translated into more than twenty languages worldwide [42]. LS can reflect one aspect of positive mental health [43] and represent a comparison judgement by comparing the respondents’ current life and internalized standards [41]. In this study, the Chinese version was revised by Qiu and Zheng [44]. The scale consists of five items measured on a seven-point Likert scale ranging from 1 (strongly disagree) to 7 (strongly agree), with a higher value indicating greater satisfaction. The Cronbach’s alpha coefficient of the SWLS in this study was 0.885. Furthermore, Kaiser–Meyer–Olkin (KMO) and Bartlett's sphericity were tested. The results showed that the KMO value was 0.846, and the significance of Bartlett's sphericity was 0.000 (*χ*^*2*^ = 7678, *p* < 0.001), which indicates that the samples satisfied the criteria for factor analysis [45].

### Health locus of control

HLC was determined with the Multidimensional Health Locus of Control Scale (MHLC) developed by Wallston et al. [16]; it has three versions: Form A, B and C, Form A and B are more general, and are identified as equivalent forms with 18 items. Form A has been most widely used in health studies [46]. Form C is a condition specific scale that is easily applicable to people with any existing medical or health-related condition [47]. The Chinese version of Form A adapted by Tong and Wang was applied in this study [48]. The scale has three dimensions, IHLC, PHLC, and CHLC, with each dimension consisting of six items rated on a 6-point Likert scale ranging from 1 (strongly disagree) to 6 (strongly agree). In the present study, the Cronbach’s alpha coefficients were higher than 0.721; the KMO values of IHLC, PHLC, and CHLC were 0.781, 0.789, and 0.780, respectively; and the significance of Bartlett's sphericity was 0.000 (*χ*^*2*^ = 2591, *p* < 0.001; *χ*^*2*^ = 2406, *p* < 0.001; *χ*^*2*^ = 3539,* p* < 0.001).

### Healthy lifestyle

In this study, healthy lifestyle was determined with the Healthy Lifestyle Questionnaire for college students designed by Jiao and Wang based on the Chinese cultural background [49]. The scale is a self-report measure consisting of 33 items and eight subscales, including exercise behaviour (EB), regular living behaviour (RLB), nutritional behaviour (NB), Unhealthy Behaviour (UB), health responsibility (HR), interpersonal support (IS), stress management (SM), and life appreciation behavior (LAB). A five-point Likert scale was used to assess each item, with a higher score representing a healthier lifestyle. In this study, the Cronbach’s alpha coefficients of the overall and eight subscales were ranged from 0.631 to 0.897. the KMO value was 0.923, and the significance of Bartlett's sphericity was 0.000 (*χ*^*2*^ = 28924, *p* < 0.001).

### Sociodemographic variables

In this study, some social demographic factors, such as age, gender, grade, race and school type, were included.

## Statistical analysis

In the present study, descriptive analysis and normal distribution tests were conducted using SPSS statistics 26. Furthermore, partial least-squares structural equation modelling (PLS-SEM) was used to analyses the data. Compared with covariance-based structural equation modelling (CB-SEM), PLS-SEM is a prediction-oriented approach to SEM requiring less statistical specification [50] and is more suitable for complex structural models that include both the formatively and reflectively measured constructs [51]. PLS-SEM is a more appropriate analytical technique for this study for the following three reasons. First, the Kolmogorov–Smirnov test results showed that major variables in the model were non-normally distributed. Thus, PLS-SEM is a more suitable tool for reducing estimation bias [52]. Second, PLS-SEM is a more recommended method for analysing complex models, and multiple interacting variables were involved in this study. Thus, PLS-SEM is a more appropriate method to estimate the model. Third, PLS-SEM allows the combination of explanatory and predictive perspectives for model estimation [53], which is in accordance with the joint consideration of this research. Therefore, SmartPLS 3.0 was applied to perform PLS-SEM.

PLS-SEM mainly focuses on the two processes of the measurement model and structural model [54]. In this study, the PLS measurement model was evaluated from three perspectives: internal consistency, convergent validity, and discriminant validity. The structural model was used to test the theoretical relationships of the hypotheses in the proposed conceptual framework. Thus, two parameters, the path coefficient (*β*) and coefficient of determination (*R*^*2*^), were measured. In addition, bootstrapping was applied to discover whether the path coefficient was significant. In addition, *Q*^*2*^ was also introduced to predict the predictive relevance.

## Results

### Sociodemographic characteristics

Table 1 presents the demographic characteristics of the participants. The majority of the participants were undergraduate college students (67.2%) and were mainly of Han nationality (91.6%). Among the participants, females (57.0%) accounted for more than half of the total; freshmen (34.4%) and sophomores (31.0%) accounted for larger proportions, while juniors and seniors accounted for 20.7% and 13.9%, respectively. Most of the participants were younger than 25 (98.8%).

**Table 1:** Demographic characteristics of the total sample (*N* = 2394) from Hubei, China during the normalization stage of COVID-19 epidemic prevention and control

	n	%
**Gender**		
Male	1029	43.0
Female	1365	57.0
**Age**		
< 20	1383	57.8
20–25	981	41.0
26–30	24	1.0
> 30	6	0.2
**Race**		
Ethnic Han	2194	91.6
Ethnic minority	200	8.4
**Grade**		
Freshman	823	34.4
Sophomore	742	31.0
Junior	495	20.7
Senior	334	13.9
**School type**		
Undergraduate college	1609	67.2
Higher vocational college	785	32.8

### Instrument validity and reliability

To evaluate the internal consistency, construct reliability was tested with the composite reliability coefficient. According to Hair [55], the minimum value should be greater than 0.7. The results showed that all of the latent constructs’ (healthy lifestyle and life satisfaction) alphas were 0.772 or above (Table 2), suggesting the reliability of the measures.

**Table 2:** Measure description and reliability of key variables from Hubei, China during the normalization stage of COVID-19 epidemic prevention and control

Scale	Items	loadings	Mean	SD	Cronbach's Alpha	CR
**SWLS**	1. In most ways my life is close to ideal	0.873	4.01	1.59	0.885	0.919
	2. The conditions of my life are excellent	0.892				
	3. I am satisfied with my life	0.916				
	4. So far, I have gotten the important things I want in life	0.831				
	5. If I could live my life over, I would change nothing	0.637				
**IHLC**	1. If my condition worsens, it is my own behavior which determines how soon I feel better again	0.605	3.85	0.75	0.732	0.798
	2. I am in control of my health	0.652				
	3. When I get sick, I am to blame	0.680				
	4. The main thing which affects my health is what I myself do	0.748				
	5. If I take care of myself, I can avoid illness	0.689				
	6. If I take the right actions, I can stay healthy	0.657				
**PHLC**	1. Having regular contact with my physician is the best way for me to avoid illness	0.671	3.33	0.77	0.721	0.798
	2. Whenever I don’t feel well, I should consult a medically trained professional	0.620				
	3. My family has a lot to do with my becoming sick or staying healthy	0.612				
	4. Health professionals control my health	0.665				
	5. When I recover from an illness, it’s usually because other people	0.721				
	6. Regarding my health, I can only do what my doctor tells me to do	0.691				
**CHLC**	1. No matter what I do, if I am going to get sick, I will get sick	0.619	2.86	0.77	0.750	0.773
	2. Most things that affect my health happen to me by accident	0.602				
	3. Luck plays a big part in determining how soon I will recover from an illness	0.771				
	4. My good health is largely a matter of good fortune	0.827				
	5. No matter what I do, I’m likely to get sick	0.739				
	6. If it’s meant to be, I will stay healthy	0.655				
**Healthy lifestyle**	**Exercise behavior**		2.37	1.05	0.764	0.865
	1. Exercise rigorously 30 min at least 3 times per week	0.870				
	2. Warm up before rigorous exercise	0.768				
	3. Have aerobic exercise (lasts for 30 to 60 min) three times a week	0.836				
	**Regular living behavior**		3.42	0.82	0.696	0.831
	1. Have daily routine	0.835				
	2. Get enough sleep every day	0.771				
	3. Have fixed meal time every day	0.760				
	**Nutrition behavior**		3.49	0.83	0.676	0.809
	1. Eat breakfast daily	0.637				
	2. Drink at least 800 cc of water daily	0.753				
	3. Include dietary fiber	0.796				
	4. Make an effort to select foods without too much oil and salt	0.676				
	**Unhealthy behavior**		1.32	0.70	0.631	0.844
	1. Drink alcohol	0.855				
	2. Smoke or use tobacco substitutes	0.855				
	**Health responsibility behavior**		3.81	0.66	0.647	0.745
	1. Follow the doctor's advice and cooperate with the treatment	0.660				
	2. Brush my teeth and use dental floss after meals	0.615				
	3. Wash hands before meals	0.630				
	4. Cover your nose and mouth when coughing or sneezing	0.711				
	5. Make an effort to maintain the public sanitation	0.708				
	**Interpersonal support behavior**		3.86	0.73	0.817	0.869
	1. Help classmates in need	0.744				
	2. Express my caring and warmth to others	0.692				
	3. Enjoy keeping in touch with relative classmates	0.804				
	4. Talk about my troubles with others	0.673				
	5. Concern about other people's feelings	0.738				
	6. Express my feelings in a way that doesn't hurt others	0.692				
	**Stress management behavior**		3.66	0.69	0.762	0.840
	1. Have own methods to reduce stress	0.666				
	2. Can accept unalterable fact in your life	0.742				
	3. Pay attention to my emotional changes	0.738				
	4. Make schedules and set priorities	0.692				
	5. Make an effort to deal with difficulties and setbacks calmly	0.743				
	**Life appreciation behavior**		3.71	0.81	0.890	0.921
	1. Willing to accept new experiences or challenges	0.796				
	2. Feel confident and optimistic about life	0.853				
	3. Make an effort to feel interesting and challenge every day	0.890				
	4. Make an effort to feel growth in a positive direction	0.888				
	5. Know the purpose of going to college	0.750				

*SD* Standard deviation, *CR* Composite reliability

Convergent validity and discriminant validity tests were conducted in this research. The convergent validity was analyse by average variance extracted (AVE) and indicator reliability. For AVE, its value should be higher than 0.5 under ideal conditions, while values between 0.36 and 0.5 can also be satisfactory [55, 56]. In this study, all the AVE values of the latent constructs met the criteria (Table 3). In terms of indicator reliability, indicator loadings at 0.40 or above are acceptable in exploratory studies [57]. The indicator loadings in this study ranged from 0.519 to 0.916. Discriminant validity test was performed by comparing the square roots of AVE and the heterotrait-monotrait ratio of correlations (HTMT). According to Fornell and Larcker, the square root of the AVE for each construct must exceed its correlation with any other constructs [58]. Table 4 presents the correlations between variables and the square roots of AVE, which confirm that the requirements are met. Moreover, the maximum value of HTMT was 0.403, which is lower than the absolute threshold of 0.9 [59]. Therefore, the discriminant validity is satisfactory.

**Table 3:** Reliability and validity of model variables (samples from Hubei, China during the normalization stage of COVID-19 epidemic prevention and control)

Variables	Cronbach’s Alpha	Composite Reliability	AVE	*Q*^*2*^
Healthy lifestyle	0.772	0.829	0.418	0.083
Life satisfaction	0.889	0.915	0.699	0.217

**Table 4:** Variable correlations and square root of average variance extracted (samples from Hubei, China during the normalization stage of COVID-19 epidemic prevention and control)

Variables	Healthy lifestyle	Life satisfaction
Healthy lifestyle	(*0.647*)	
Life satisfaction	0.527^***^	(*0.836*)

Italic values in the parentheses are square roots of AVE

Multicollinearity was evaluated by using the variance inflation factor (VIF). According to Lee et al. [60], the recommended threshold value must be lower than 3.3. In this study, the highest VIF was 2.608, indicating that there was no multicollinearity problem. Additionally, the predictive relevance* Q*^*2*^ was calculated with the blindfolding procedure. As shown in Table 3, all values of *Q*^*2*^ were above zero, which supports the sufficient predictive relevance of this model. Finally, *R*^*2*^ was used to determine the explanatory power of a structural model. According to Höck [61] and Hair [55], coefficient of determination (*R*^*2*^) values of 0.25, 0.50 and 0.75 are generally considered as weak, medium and large effects, respectively. The *R*^*2*^ values in this study were 0.150 and 0.289, which represent weak and medium effects, respectively.

### Path analysis

The PLS algorithm and bootstrapping method with 5000 subsamples were applied to estimate the hypothesized path coefficient and its validity.

Analysis of the total effects revealed that IHLC and PHLC were positively and significantly related to LS (*β* = 0.170, t = 7.171, *p* < 0.001, 95% CI [0.121; 0.215]; *β* = 0.205, t = 7.539, *p* < 0.001, 95% CI [0.166; 0.269]), while CHLC had a significant negative correlation with LS (*β* = –0.148, t = 5.953, *p* < 0.001, 95% CI [–0.196; –0.098]). Thus, H1, H2, and H3 are supported.

**Fig. 1:**
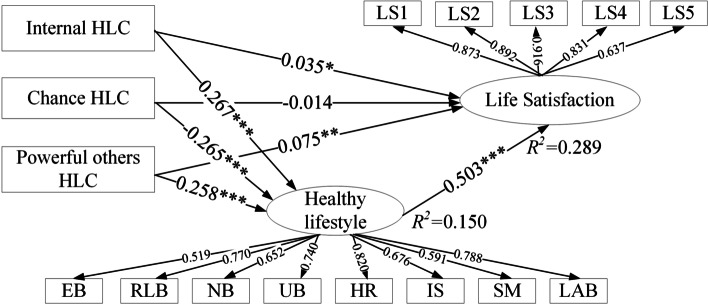
The structural model consisted of health locus of control, healthy lifestyle and life satisfaction of Chinese college students in Hubei, China during the normalization stage of COVID-19 epidemic prevention and control. Control variables: gender, grade, race, school type; HLC health locus of control, LS life satisfaction, EB exercise behavior, RLB regular living behavior, NB nutrition behavior, UB unhealthy behavior, HR health responsibility, IS interpersonal support, SM stress management, LAB life appreciation behavior. * < 0.1, ** < 0.05, *** < 0.01

The results of the path coefficient (Table 5 and Fig. 1) revealed that IHLC was significantly and positively related to healthy lifestyle (*β* = 0.267, t = 12.254, *p* < 0.001, 95% CI [0.225; 0.310]). PHLC also showed a significant positive correlation with healthy lifetyle (*β* = 0.258, t = 10.005, *p* < 0.001, 95% CI [0.206; 0.308]). CHLC was significantly and negatively related to healthy lifestyle (*β* = –0.265, t = 10.789, *p* < 0.001, 95% CI [–0.314; –0.215]), and there was a significant positive correlation between healthy lifestyle and LS (*β* = 0.503, t = 28.313, *p* < 0.001, 95% CI [0.467; 0.539]). H4, H5, H6, and H7 are confirmed.

**Table 5:** Path coefficients of the structural model and the mediation effect of healthy lifestyle on the relationship between health locus of control and life satisfaction among Chinese college students (*N* = 2394) in Hubei, China during the normalization stage of COVID-19 epidemic prevention and control

Path	Path Coefficients (*β*)	t	*P*-value	CI (2.5%)	CI (97.5%)
IHLC → LS	0.035	2.062	0.097	-0.005	0.078
PHLC → LS	0.075	3.102	0.002	0.102	0.158
CHLC → LS	-0.014	0.623	0.533	-0.059	0.028
IHLC → Healthy lifestyle	0.267	12.254	< 0.001	0.225	0.310
PHLC → Healthy lifestyle	0.258	10.005	< 0.001	0.206	0.308
CHLC → Healthy lifestyle	-0.265	10.789	< 0.001	-0.314	-0.215
Healthy lifestyle → LS	0.503	28.313	< 0.001	0.467	0.539
IHLC → Healthy lifestyle → LS	0.135	11.380	< 0.001	0.110	0.157
PHLC → Healthy lifestyle → LS	0.130	9.263	< 0.001	0.102	0.156
CHLC → Healthy lifestyle → LS	-0.134	9.844	< 0.001	-0.160	-0.107

*IHLC* Internal health locus of control, *PHLC* Powerful others health locus of control, *CHLC* Chance health locus of control, *LS* Life satisfaction

### The mediating effect of healthy lifestyle

Healthy lifestyle positively mediated the linkage between IHLC and LS (*β* = 0.135, t = 11.380, *p* < 0.001, 95% CI [0.110; 0.157]), as well as that between PHLC and LS (*β* = 0.130, t = 9.263, *p* < 0.001, 95% CI [0.102; 0.156]). Healthy lifestyle negatively mediated the linkage between CHLC and LS (*β* = –0.134, t = 9.844, *p* < 0.001, 95% CI [–0.160; –0.107]), supporting H8. According to Sarstedt [51], a variance accounted for (VAF) value lower than 20% indicates no mediating effect; a value between 20 and 80% represents partial mediation; and a value higher than 80% indicates full mediation. Thus, healthy lifestyle partially mediated the association of IHLC and PHLC with LS (VAF_(IHLC)_ = 79.412%; VAF_(PHLC)_ = 63.415%), and fully mediated the linkage between CHLC and LS (VAF_(CHLC)_ = 90.540%).

## Discussion

The present study examined the correlations between three dimensions of HLC and LS among college students in Hubei Province during the normalization stage of COVID-19 pandemic prevention and control, and further explored the mediating effect of healthy lifestyle. In line with previous research, this study revealed that LS is positively related to IHLC, and negatively associated with CHLC [19, 62]. Specifically, college students with higher IHLC tend to have higher LS than those with higher CHLC. These results imply that when faced with negative life events such as COVID-19 health problems, pressure, low grades and financial difficulties, college students with higher IHLC tend to adopt problem-solving strategies, while those with higher CHLC are inclined to respond to them with emotional reactions such as sadness or anger [63]. Thus, college students with higher IHLC may overcome their annoyance and live a happy life [64]. However, college students with higher CHLC tend to believe that their success is dependent on chance, fortune and destiny [19, 65]. They also tend to carry their burdens into the future and constantly feel disappointed, thereby having more difficulties forming a feeling of well-being and LS [64].

In contrast to previous findings [20, 25, 62], PHLC was positively related to LS among the college students in this study. The reason for this discrepancy might be the differences between individualist-and-collectivist cultures. Most previous studies were conducted in an individualistic cultural context, such as the United States or European countries. People in these cultures are more likely to emphasize the personal self, whereas people from collectivist cultural backgrounds, such as Asia, South America, and Africa, are more inclined to emphasize the collective self, namely connections with groups [66, 67]. Moreover, in collectivist cultures, individuals tend to trust powerful others such as professors, doctors and counsellors and are motivated by the judgements and directions of those in power [68]. Collectivism is deeply rooted in Chinese culture. Therefore, when faced with health risks, individuals, including college students are more likely to turn to significant others, such as physicians, professors, counsellors, family members, and peers [68].

Given its mediating role, healthy lifestyle may transmit the effect of the three types of HLC on LS. Partially in accordance with previous findings, healthy lifestyle was positively correlated with IHLC but negatively correlated with CHLC. College students with higher IHLC are more willing to take responsibility and believe that their health is determined by themselves rather than by fortune or chance [69]. Thus, this group of college students is more willing to be engaged in health-promoting behaviour, such as physical exercise and healthy dieting, which are essential for developing a healthy lifestyle and promoting LS [20, 70]. Conversely, college students with higher CHLC believe that their health is dependent on destiny, luck and chance [19] and tend to have potential risks of smoking, drinking, eating disorders, depression, and anxiety [20], which reduce their possibility of actively participating in healthy lifestyles and therefore result in lower LS. Notably, PHLC positively predicted healthy lifestyle in this study, which is consistent with the findings in a collectivist culture background [68, 69]. This finding indirectly verifies the viewpoint mentioned above: when faced with public health emergencies, college students from collectivist cultures tend to rely on role models such as health professionals, counsellors, family members, peers and other significant people, who have great impacts on their feelings, thinking and behaviours [71].

## Implications

There is ample evidence that during COVID-19 pandemic, college students may suffer social isolation, uncertainty, online study pressure, or even anxiety, depression, and general mental distress [2–4]. Students with higher IHLC reported that the pandemic was easier to bear [2] and remained motivated to develop a healthier lifestyle to combat the epidemic. Powerful others also play a positive role in guiding students’ attitudes towards life. Therefore, the following considerations may also have certain reference values in future pandemics.

Given that the locus of control changes over time, particularly in the face of health issues [72], improving of IHLC should be considered. In addition, the combination of universal and targeted intervention measures should be adopted. For ordinary college students, psychological health courses and physical education courses should included in core college curricula. Thus, first, it is necessary to give full play to the educational and guiding role of teachers in these courses, and integrate positive psychology into the teaching process to cultivate positive thinking and emotion as well as promote the IHLC of college students. Second, more emphasis should be placed on the role of authorities and examples [73]. On one hand, authorities such as health experts and doctors can be invited to give lectures and lead serial thematic educational activities to promote rational conceptions of health among college students. On the other hand, colleges should help students establish a correct health viewpoint by setting peer examples, particularly psychologically positive students with a healthy lifestyle and high responsibility for their own health. The notion of healthy living can be popularized through poster campaigns, tweets in official WeChat accounts, peer influence and other methods. In addition, the formation of the locus of control starts in the family, and personal health values are also greatly shaped by family members’ beliefs, attitudes and behaviours [69]. Therefore, intervention measures can be started earlier, and community-based health service centres can play a positive role in shaping the health beliefs of family members.

College students with unhealthy lifestyles, high CHLC and low LS, should first be precisely identified. Specifically, they can be identified through systematic appraisal during the “Mental Health of College Students” curriculum. In particular, Chinese colleges generally offer the core curriculum “Mental Health of College Students” for students; thus, their healthy habits, locus of control and feelings of life can be discovered through psychological practical activity, psychology test games, and final curriculum examination. Beyond that, the students can also receive individual counselling from professional psychological consultants. Once these students are identified, psychological counselors can apply cognitive behaviour group therapy for targeted intervention, and help them improve their self-reflection, positive thinking, optimism and self-confidence [19]. In this way, a mutual effect mechanism among college students can be built, thereby making it easier to develop a healthy lifestyle.

## Limitations

Notably, this study has some limitations. First, the survey was conducted with the purposive sampling method, which may cause representativeness bias. Since the study samples were collected only from six colleges in Hubei Province, the findings of the present study may have some limitations in generalizability. Furthermore, since it is a cross-sectional study, we could obtain only correlations and could not reveal the causal relationships among the variables. Therefore, large-scale longitudinal research should be conducted to further explore the causal relationship among variables in future studies.

## Conclusions

In this study, healthy lifestyle and LS were found to be significantly and positively associated with IHLC and PHLC but to have significant negative correlations with CHLC under collectivist cultural background. Additionally, healthy lifestyle significantly mediates the relationship between HLC and LS. These results suggest that HLC is crucial to the formation of a healthy lifestyle and improving LS in college students, and a healthy lifestyle is a vital factor for LS. Thus, enhancement of IHLC, promotion of the health guiding role of authorities and role models, and development of a healthy lifestyle may be key routes for improving the LS of college students.

## Supplementary Information

**Additional file 1. **

## Data Availability

The datasets applied during the current study are available from the corresponding author on reasonable request.

## Abbreviations

HLC: Health Locus of Control
IHLC: Internal Health Locus of Control
CHLC: Chance Health Locus of Control
PHLC: Powerful Others Health Locus of Control
EB: Exercise Behavior
RLB: Regular Living Behavior
NB: Nutritional Behavior
UB: Unhealthy Behavior
HR: Health Responsibility
IS: Interpersonal Support
SM: Stress Management
LAB: Life Appreciation Behavior
LS: Life Satisfaction
PLS-SEM: Partial Least Squares Structural Equation Modelling
AVE: Average Variance Extracted
HTMT: Heterotrait-monotrait Ratio of Correlations
VIF: Variance Inflation Factor
VAF: Variance Accounted For value
