# Supplementary material for: Healthy lifestyle mediates the association between health locus of control and life satisfaction among college students in Hubei, China: during the normalization stage of COVID-19 epidemic prevention and control
Source: Arch Public Health. 2023 Jul 24;81:136. doi: 10.1186/s13690-023-01145-9 (PMC10364408; doi:10.1186/s13690-023-01145-9)
Supplement: Supplementary file 1 — Additional file 1. [file 13690_2023_1145_MOESM1_ESM.pdf]

What is known?

Life satisfaction is a cognitive dimension of subjective well-being, and has been identified as a predictor and indicator of functioning, presence of comorbidities and clinical symptoms. It showed a significant decrease under the influence of the COVID-19 pandemic. In order to explore its influencing mechanisms, many studies have been discussed in terms of cognitive or behavioral dimension. Thus, the direct relationships between health locus of control (cognitive dimension), healthy lifestyle (behavioral dimension) and life satisfaction has been investigated. To be specific, the positive relationship between internal locus of control (IHLC) and life satisfaction, and the negative relationship between chance health locus of control (CHLC) and life satisfaction were discovered. Whereas, both positive and negative relationships between powerful others locus of control (PHLC) were found, thus, this relationship still need further investigation. Moreover, there have been few studies to verify the mediating role of healthy lifestyle between locus of control and life satisfaction.

What does the study adds?

This study incorporates the locus of control (cognitive dimension) and healthy lifestyle (behavioral dimension) into one model, and verified the correlations of IHLC, CHLC, PHLC with life satisfaction under the collectivism culture background. Further, the mediating role of healthy lifestyle was also proved. In addition, this study focused on a relatively more psychologically vulnerable group----college students, and a total of 2394 college students from six colleges in Hubei Province were participated. Partial least squares structural equation modelling (PLS-SEM) was employed to analyze the relationships between three dimensions of health locus of control, healthy lifestyle, and life satisfaction. Bootstrapping method was applied to verify the mediation effect of healthy lifestyle.

The findings found that healthy lifestyle and life satisfaction have positive correlations with both IHLC and PHLC, but have significant negative correlations with CHLC. Further, healthy lifestyle mediated the relationships of IHLC, PHLC and CHLC with life satisfaction, which means the health locus of control not only directly related to life satisfaction, but also indirectly related to it through healthy lifestyle. These outcomes indicates that for college students, improvement of IHLC, promotion of the health guiding role of authorities and role models, and development of healthy lifestyles may

be key routes to improve their life satisfaction.

What are implications for clinical practice, public health and / or research?

The conclusions may have some implications and suggestions for college students' health educations. First, psychological health courses and physical education courses should be listed in the core curriculums in college. Second, colleges should help students establish a correct health viewpoint by setting peer examples and popularizing healthy notions. Third, since the formation of locus of control starts in family, the community-based health service centers can play a positive role in shaping the health belief of family members. In addition, for those college students with unhealthy lifestyles, high CHLC and low life satisfaction, they should first be precisely identified through systematic appraisal or individual conversation with college counselors. Once these students are identified, psychological counselors can apply the cognitive behavioral group therapy for targeted intervention, and help them improve their self-reflection, positive thinking, optimism and self-confidence, which can in turn improve their life satisfaction.
